# Research progress of the SLFN family in malignant tumors

**DOI:** 10.3389/fonc.2024.1468484

**Published:** 2024-11-04

**Authors:** Jiale Yu, Zhijuan Guo, Junyi Zhang

**Affiliations:** ^1^ Inner Mongolia Medical University, Hohhot, China; ^2^ School of Basic Medicine, Chifeng University, Chifeng, China; ^3^ Department of Pathology, Peking University Cancer Hospital & Affiliated Cancer Hospital of Inner Mongolia Medical University, Hohhot, China

**Keywords:** SLFN, tumors, immune infiltration, therapy, immunosuppression

## Abstract

The Schlafen (SLFN) gene family has emerged as a critical subject of study in recent years, given its involvement in an array of cellular functions such as proliferation, differentiation, immune responses, viral infection inhibition, and DNA replication. Additionally, SLFN genes are linked to chemosensitivity, playing a pivotal role in treating malignant tumors. Human SLFNs comprise three domains: the N-terminal, middle (M), and C-terminal. The N- and C-terminal domains demonstrate nuclease and helicase/ATPase activities, respectively. Meanwhile, the M-domain likely functions as a linker that connects the enzymatic domains of the N- and C-terminals and may engage in interactions with other proteins. This paper aims to present a comprehensive overview of the SLFN family’s structure and sequence, examine its significance in various tumors, and explore its connection with immune infiltrating cells and immune checkpoints. The objective is to assess the potential of SLFNs as vital targets in cancer therapy and propose novel strategies for combined treatment approaches.

## 1 Article

The mortality rate of malignant tumors remains among the highest globally, representing a significant global issue. Although early cancer screening has helped reduce the incidence of certain cancers, such as cervical cancer, the overall impact varies. According to the American Cancer Society, the incidence of cervical cancer dropped by 65% within seven years among the first cohort vaccinated against human papillomavirus (HPV) (1). In contrast, the benefits of early screening and prevention for digestive system tumors are less pronounced due to their nonspecific symptoms and low early diagnosis rates. This often results in missed opportunities for early intervention and treatment. Traditional treatment modalities such as surgical excision, radiation therapy, and chemotherapeutic treatment can be unproductive or poorly tolerated in some patients, especially considering individual differences and varying tumor stages. Although molecular targeted drugs like trastuzumab and apatinib have been approved for gastric cancer, the progress in targeted therapy for gastric cancer lags significantly behind that for lung and breast cancers (2, 3). Immunotherapy, which enhances the body’s immune response against tumors by activating or mobilizing the immune system, has garnered extensive clinical attention as a promising treatment approach. The Schlafen (SLFN) gene family, which belongs to the gene cluster in vertebrates, produces proteins that show considerable sequence resemblance and exhibits differential expression across various tissues and species. These genes are extensively produced in tumor cells and are essential for the differentiation of immune cells as well as the regulation of immune responses. The differential expression of this factor in tumors suggests its potential as a serological biomarker for preoperative diagnosis.

Additionally, its involvement in immune regulation, particularly in inhibiting immune evasion, offers promising new avenues for immunotherapy. These aspects have been extensively explored in relevant studies (4). However, the regulatory effects of the SLFN family are not always positive. Therefore, further investigation into their roles in different diseases is crucial to improve therapeutic outcomes in cancer treatment.

## 2 Overview of the SLFN groups of genes

### 2.1 Individuals within the SLFN family

First identified by Schwarz in 1988 in mice, the SLFN gene family derives its name from the the German term ‘Schlafen,’ which means ‘slumber’ in English, reflecting the initial finding that Schlafen proteins halt cell division. In humans, this family consists of SLFN5, SLFN11, SLFN12, SLFN12L, SLFN13, and SLFN14 genes (5).

The SLFN genes represent a group of evolutionarily conserved genes present across various vertebrate species (6). They participate in numerous biological functions, including cell proliferation, differentiation, immune responses, viral pathology inhibition, and DNA duplication, and play a crucial role in chemo-responsiveness (7). SLFN intracellular localization proteins differs: in mice, SLFN proteins predominantly reside in the cytoplasm, while in humans, SLFN12 and SLFN13 are found in the cytoplasm, while the localization of SLFN11, SLFN14, and SLFN5 remains uncertain. These localizations can be identified using immunostaining and immunofluorescence methods (8). Transcriptional data from the CCLE (Cancer Cell Line Encyclopedia) repository (9) show that every SLFN gene is independently transcribed. SLFN11, SLFN5, SLFN13, and SLFN12 are broadly transcribed in cancer cells, while SLFN12L, SLFN14, and SLFNL1 display reduced levels of expression (**Table 1**).

**Table 1:** The association of Schlafen with diseases, chemotherapy, and the primary pathways involved.

		SLFN2	SLFN3	SLFN5	SLFN11	SLFN12	SLFN13
Digestive system tumors	Gastric cancer			√	√		
	Colorectal cancer		√	√	√	√	
	Liver cancer				√		
	Pancreatic cancer			√			√
Respiratory system tumors	Lung cancer			√	√	√	√
Urological system tumors	Renal cancer						√
Other systemic tumorsor diseases	Prostatic cancer			√	√	√	
	Glioblastoma			√			√
	Breast cancer			√	√	√	√
	HIV			√	√	√	√
	Malignant melanoma	√	√				
Chemotherapy sensitivity					Exist	Exist	
Signaling Pathway				AKT/gsk-3β/β- catenin	Notch		

The meaning of √ represents the differential expression of the protein/gene in a certain disease, which is related to the disease process.

### 2.2 Structure of SLFN proteins

**Figure 1:**
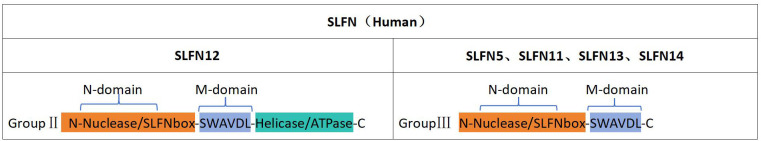
Classification of Human SLFN Genes and Proteins.

SLFN proteins can be grouped into three distinct categories determined by their structural traits as well as functional fields (10). The first category comprises proteins with a shared N-terminal domain featuring nuclease-like structures and a conserved SLFN motif found within every SLFN protein. The second category includes proteins that, in addition to the N-terminus, possess an intermediate linker region (M-region) containing the SWAVDL pattern and regions potentially involved in protein interactions. The third and largest category encompasses proteins with a functional helicase/ATPase domain in the C-terminal region, defined through the existence of Walker A/B motifs (11). The human SLFN gene produces polypeptides that are classified solely into group II (SLFN12) and group III (SLFN5, SLFN11, SLFN13, and SLFN14) (**Figure 1**).

The N-terminal section of SLFN family members is a pivotal structural component linked to tRNA/rRNA endonuclease activity (12). The structure of the SLFN-N domain is preserved in both SLFN12 and SLFN5, with minor conformational variations (13, 14). SLFN14 interacts with ribosomes through its M-domain, and modifications in the M-domain diminish endoribonuclease activity in the nucleus (15). This implies that the M-region in SLFN family members may serve as a binding site for nucleotides or active cofactors. the M-domain in SLFNs might act as a docking site for nucleic acids or functional cofactors. SLFN11 is capable of suppressing the translation of viral proteins during HIV infection by cleaving specific tRNAs, indicating a link between SLFN proteins and immunity. Furthermore, the ATPase function of SLFN11 is essential for eliminating cancer cells with replicating DNA damage and open chromatin, resulting in fatal replication pause and the induction of stress response genes within the FOS-JUN pathway (16). Additionally, the monkeypox virus has been shown to carry a virulence factor with a single SLFN domain (17). Although the role of this domain in virulence has not been evaluated, its involvement in controlling host-pathogen interactions seems plausible. Lastly, the SLFN-associated fold, known as the Smr domain, has been shown to function as a nuclease in RNA quality control mechanisms (18). A recent research conducted by Nadezda Podvalnaya and her collaborators, published in Nature, examined the role of trimeric schlafen domain nucleases in the processing of PIWI-interacting RNAs (piRNAs) (19). Collectively, these activities propose that the SLFN-like domain serves a significantly preserved function in immunity and stress-related mechanisms. Research has shown that SLFN domains can form polymeric complexes, which may reveal highly specific nucleolytic activity. It is conceivable that proteins incorporating SLFN-associated folds may generate highly specific enzymes that help organisms defend against infectious nucleic acids.

### 2.3 Association of the SLFN group with immune cell infiltration

**Figure 2:**
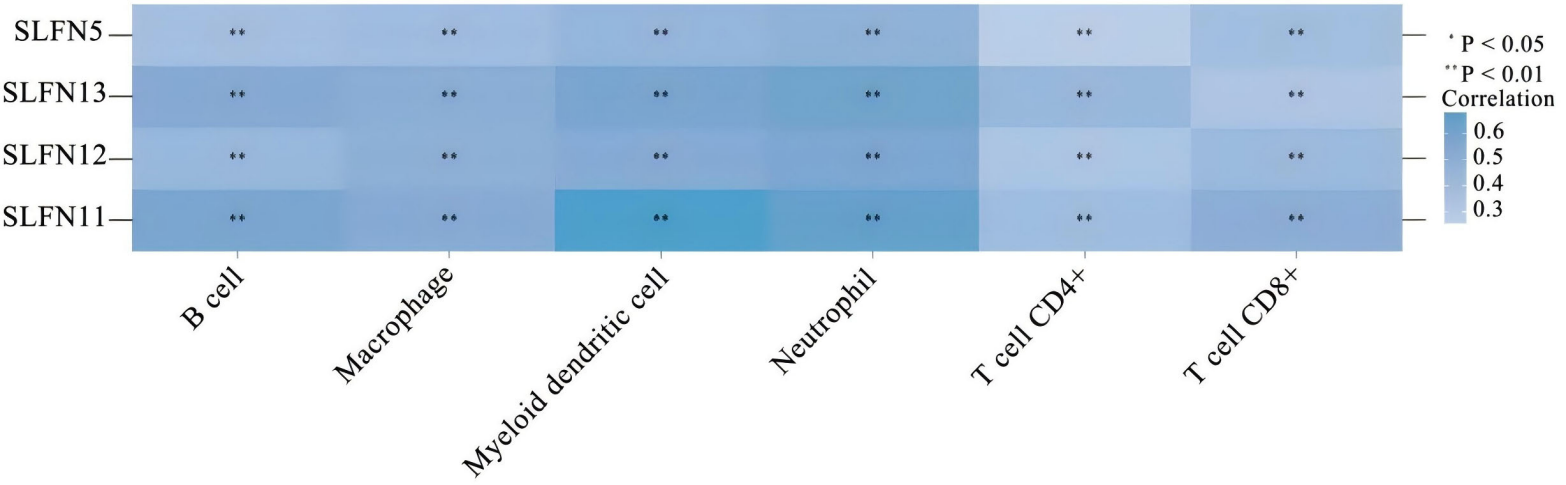
The correlation between widely expressed SLFN family genes in cancer and immune-related factors. Darker colors represent stronger correlations. *p < 0.05, **p < 0.01.

**Figure 3:**
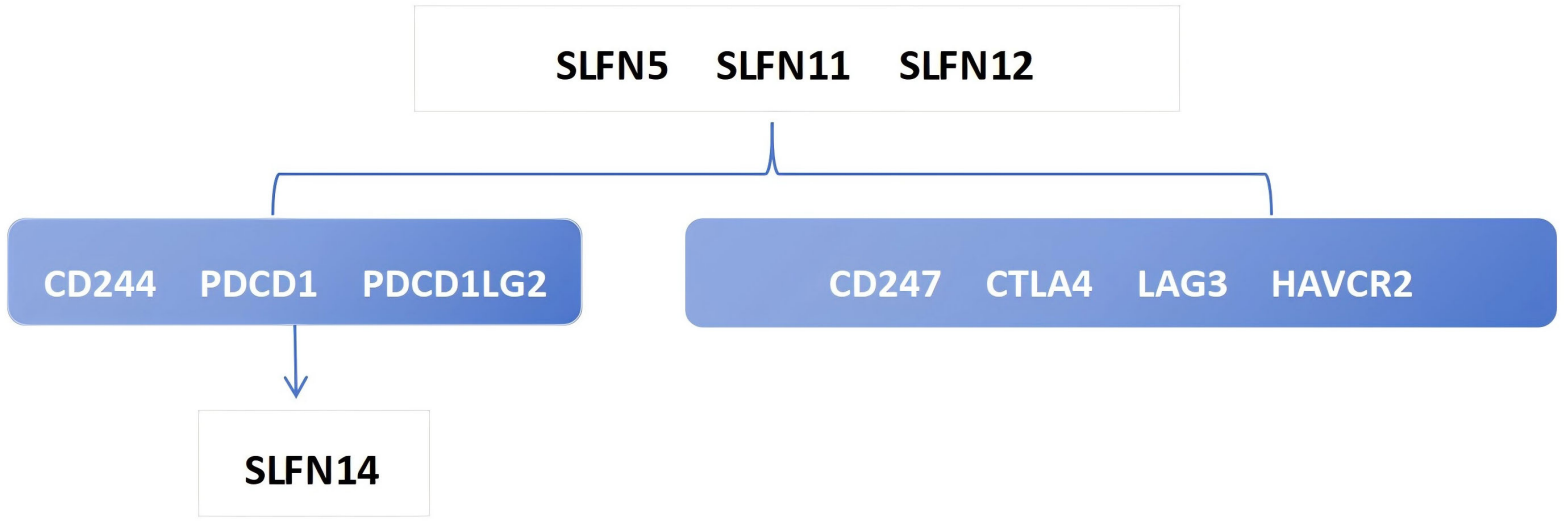
The figure illustrates the relationship between schlafen and immune checkpoints.

The heterogeneity of tumors in terms of their invasive capacity, growth rate, and drug sensitivity presents significant challenges for treatment. However, it is well-recognized that tumors engage in continuous, dynamic interactions with their microenvironment. Stromal and immune cells, as crucial elements of the tumor-associated microenvironment (TME), have significant impacts on tumor advancement and therapeutic responses. The cells involved in shaping the tumor immune microenvironment are referred to as tumor-infiltrating immune cells (TIICs), which primarily include macrophages, lymphocytes, fibroblasts, and myeloid-derived suppressor cells. Among these, CD8+ T cells are the most essential for anti-tumor activity in the TME, as they cause tumor cell death through the secretion of cytokines, including IFN-γ. However, TIICs may also promote tumor progression during initiation, development, and metastasis stages (20). Thus, TIICs within the TME can serve as markers for evaluating the efficacy of immunotherapy (21, 22). We examined the relationship between SLFN family members and immune cell types utilizing data obtained from The Cancer Genome Atlas (TCGA) (**Figure 2**). The analysis shows that the previously discussed widely expressed SLFN family members at the transcriptional level in cancers are correlated with TIICs. Among them, the association between SLFN11 and dendritic cells as well as CD8+T cells was significantly enhanced. Of particular note is that the correlation between various members of Schlafen and macrophages is generally and consistently strong. Given the crucial role played by CD8+T cells and macrophages in immune infiltration, this provides a clearer target for our subsequent research. Previous studies, including the detailed exploration of The association between the SLFN gene family and immune cell infiltration in gastric carcinoma, have demonstrated positive correlations between the levels of SLFN5, SLFN11, SLFN12, and SLFN12L expression, and SLFN14 in the context of immune infiltration involving CD8+ T cells, CD4+ T cells, macrophages, neutrophilic granulocytes, and antigen-presenting cells (23). Therefore, it is anticipated that certain members of the SLFN family may promote the proliferation of gastric cancer by increasing immune cell infiltration. During the initial phases of tumorigenesis, immune cells with anti-tumor functions tend to destroy tumor cells, but eventually, neoplastic cells can evade immune surveillance occurs via multiple mechanisms and can also suppress the cytotoxic effects of immune cells. One such mechanism of immune evasion is the production of immune checkpoint molecules by neoplastic, such as the interaction between PD-1-expressing T cells and their corresponding PD-L1 or PD-L2 ligands present on the surface of neoplastic cells, leading to T cell inactivation and an inability to kill tumor cells (24). Currently identified immune checkpoints comprise various molecules such as CTLA-4, PD-1, TIM-3, and LAG-3, among others. Research has shown that the levels of SLFN5, SLFN11, and SLFN12 expression are positively associated with CTLA-4 et al (23) (**Figure 3**). Thus, the SLFN family not just indicates infiltration of immune effector cells in tumors,but also functions as a predictive factor for expression of immune regulatory checkpoints, implying its potential role as a biomarker or therapeutic target in immunotherapy for gastric cancer.

As a crucial ecosystem in tumor development, M1 macrophages exhibit an anti-tumor, pro-inflammatory phenotype, while M2 macrophages play an immunoregulatory role by promoting immunosuppression and tumor progression, making them a focal point of tumor immunology research (25). Significant progress has been made in understanding this relationship through the work of Professor Ning Ren’s team at Fudan University. Their research demonstrated that SLFN11 is significantly upregulated in tumors responding to immune checkpoint inhibitors (ICIs). Tumor-specific deficiency of SLFN11 enhanced the infiltration of Suppressive macrophages and exacerbated the progress of hepatocellular carcinoma (HCC). This effect was mediated by SLFN11 deficiency inducing the transcription and secretion of CCL2 in liver cancer cells through the Notch pathway. Knockdown of SLFN11 in HCC cells facilitated macrophage movement and M2-like differentiation in a manner dependent on CCL2, which subsequently increased PD-L1 expression through the activation of the NF-κB signaling pathway. Blocking CCL2 signaling and M2 macrophage polarization in SLFN11-deficient tumors improved the efficacy of anti-PD1 therapy for HCC (4). SLFN11 thus holds potential as a biomarker in peripheral blood for forecasting and continuously assessing the response to immune checkpoint inhibitors (ICIs) in advanced stages liver cancer via as a biomarker in peripheral blood for forecasting and continuously assessing the response to immune checkpoint inhibitors (ICIs) in advanced liver cancer via employing minimal invasiveness techniques, providing great promise for clinical translation. The article proposes that CCL2-regulated macrophage infiltration and foster a nuanced M2-type polarization, together with the increased expression of the presence of Programmed Death Ligand 1 (PD-L1) in tumor cells, may be the underlying mechanisms through which deficiency in SLFN11 promotes immune escape in liver cancer.

The article suggests that CCL2-dependent macrophage infiltration and M2-like polarization, along with the upregulation of PD-L1 in tumor cells, represent the potential mechanisms by which SLFN11 deficiency facilitates immune evasion in liver cancer.

In a study by Alexander Puck et al. on primary human immune cells, it was found that the upregulation of SLFN genes is primarily dependent on autocrine type I interferon signaling. The swift decline in SLFN expression after T-cell receptor activation suggests that SLFN plays a crucial role in preserving the quiescence of human T-cells (26). SLFN4 is also upregulated during macrophage activation (27). Further research by Edoardo Isnaldi et al. revealed that elevated levels of SLFN11 expression in breast cancer is associated with increased invasiveness and immunoactivated tumors, in contrast, lack of significant SLFN11 expression is associated with tumors that are less aggressive and exhibit immunosuppressive characteristics (28). Additionally, SLFN5 was shown to co-localize in conjunction with T cells and type 2 macrophages in precancerous stomach lesions, indicating an immunosuppressive function of SLFN5 in gastric cancer (29). Therefore, interventions targeting the immune microenvironment may offer a breakthrough in maximizing therapeutic efficacy. Thus, the SLFN gene family’s association with immune cell infiltration within tumors, along with its role as a predictive indicator for immune checkpoint expression, underscores its potential as a valuable biomarker or therapeutic focus in the realm of gastric cancer immunotherapy.

## 3 SLFN family and disease association

The SLFN family is linked to the initiation and advancement of multiple types of tumors (**Table 2**). This table covers common clinical systemic malignancies, including lung cancer, gastrointestinal tumors, and breast cancer. Below, we will provide a brief overview of the role of each protein in these diseases.

**Table 2:** The role of SLFN family members in disease progression.

	Proposed Mechanism
SLFN5	Prevention of epithelial-mesenchymal transition(EMT)in breast cancer (30) Is inversely associated with tumor growth in human BRCA (31) Inhibition of HIV-1 transcription by epigenetics (32) New therapeutic targets for pancreatic cancer (33) Enhance the malignant degree of glioblastoma (34) Reduce lung damage in pneumonia (35)
SLFN11	Inhibit HIV-1 virus replication (36)
	It is expected to be a biological marker for lung cancer and liver cancer (4, 37) Increased sensitivity to chemotherapy (38. 39, 40)
SLFN12	To improve the efficacy of radiotherapy and chemotherapy for triple negative breast cancer (41, 42)
	Improve the prognosis of lung adenocarcinoma and identify it as a therapeutic target (43)
SLFN13	Increase the body's resistance to HIV-1 (8) Improve the ability to resist glioblastoma (44)

### 3.1 Associations of SLFN5 with tumors

Studies have shown that SLFN5, as a transcriptional repressor, is expressed in breast cancer (BRCA) and plays a role in preventing epithelial-mesenchymal transition (EMT) (30), a key pathological process in tumor progression. This has been confirmed to be regulated through the β-catenin signaling pathway (31, 45). Additionally, SLFN5 preserves and reestablishes the epithelial phenotype in BRCA cells via transcriptional downregulation of ZEB1 (46). The SLFN5 expression exhibits a negative correlation with tumor progression in human BRCA. Lentiviral overexpression of SLFN5 in BRCA cell lines inhibited tumorigenicity in nude mice. Both knockout and overexpression studies of SLFN5 in BRCA cell lines have demonstrated that SLFN5 suppresses cell proliferation and colony formation, while promoting apoptosis by upregulating the transcription of the PTEN gene, located on chromosome 10 and known for its role as a tumor suppressor, holds significant importance in oncological contexts. This results in molecularing changes in the downstream AKT pathway, affecting proliferation and apoptosis (31). Thus, SLFN5 could potentially be exploited as a therapeutic intervention for BRCA-related conditions.

In Jiwei Ding’s research on HIV-1, SLFN5 was identified as a mechanism that inhibits HIV-1 transcription through epigenetic regulation potentially acting as a key determinant of HIV-1 latency (32). Furthermore, given the rising incidence of pneumonia in recent years, researchers have found that in a model of pneumonia induced by lipopolysaccharide (LPS), the knockout of SLFN5 mitigates LPS-triggered lung injury by modulating the JAK/STAT pathway (35).

Regarding the treatment of human pancreatic cancer, the team led by Mariafausta Fischietti identified SLFN5 as a pivotal regulator of cellular S-phase progression within the cell cycle through its binding/blocking of the transcriptional repressor E2F7, suggesting SLFN5 emerges as a promising novel therapeutic focus for pancreatic malignancies (33). In a separate research endeavor by Ahmed et al. (34), it was demonstrated that the expression SLFN5 is known to enhance cellular motility and aggressiveness of glioblastoma cells, and elevated SLFN5 expression is significantly correlated with the presence of high-grade gliomas and poor patient survival, thereby driving the malignancy of the disease.

In addition to its role in tumors, SLFN5 has been recognized as a restriction factor for herpes simplex virus (HSV), acting to inhibit viral transcription (47). Androgen deprivation therapy (ADT) is a potent therapy for managing prostate malignancy, nevertheless, the majority of patients ultimately progress to Chronic, treatment-resistant prostate cancer with a lethal prognosis (CRPC). The protein SLFN5 has been recognized as an androgen receptor-regulated entity in castration-resistant prostate cancer (CRPC). A correlation exists between elevated levels of SLFN5 in CRPC tumors and diminished patient prognosis (48). Moreover, SLFN5 functions as a new regulator of LAT1, an important facilitating optimal biochemical function, and significantly influences mTORC1 activity in chemotherapy-resistant metastatic prostate carcinoma.

### 3.2 Association of SLFN11 with tumors

SLFN11 is an antiviral restriction factor induced by interferon, exhibiting both tRNA endoribonuclease and DNA-binding activities (36). Under replication stress, it is deployed to arrest stalled replication forks, thereby preventing the replication of select viruses, including Human Immunodeficiency Virus 1 (HIV-1), through the regulation of the transfer RNA pool. Chenhao Zhou et al. demonstrated through experiments that SLFN11 targets RPS4X via the MTR signaling pathway, suggesting its significance in suppressing hepatocellular carcinoma (HCC) initiation and metastasis (49). Furthermore, low SLFN11 expression in HCC correlates with decreased overall survival and increased risk of recurrence, making it an independent prognostic factor for HCC patients. Non-invasive detection of circulating tumor cells (CTCs) offers a valuable complement to tissue-based techniques such as immunohistochemistry (IHC) and has the distinct advantage of allowing longitudinal monitoring. Dynamic expression of SLFN11 in CTCs from small-cell lung cancer (SCLC) patients as a liquid biomarker provides a viable alternative to biopsy for SCLC detection (37). Additionally, a study by Professor Ning Ren’s team at Zhongshan Hospital, Fudan University, found a statistically significant positive correlation has been observed between elevated serum levels of SLFN11 protein and the therapeutic efficacy of immune checkpoint blockade agents in the treatment of hepatocellular carcinoma (HCC) (4). While SLFN11 is involved in disease progression, much of the current research focuses on its therapeutic implications (38, 39). SLFN11 sensitizes cells to chemotherapeutic agents by preventing DNA damage repair (40), including cisplatin, carboplatin, irinotecan, mitoxantrone, and cytarabine. This raises the question of whether upregulating SLFN11 expression could reverse chemoresistance in tumors with low SLFN11 expression. Numerous studies have substantiated this hypothesis (50–53), which clearly illustrated that restoring expression levels of SLFN11 through epigenetic reversal of hypermethylation resensitized cells to chemotherapy. Chemotherapeutic compounds targeting Topoisomerase I (TOP1), such as irinotecan and its metabolite SN-38, induce double-stranded DNA damage as a key mechanism of their action. Numerous research findings indicate a significant correlation between elevated SLFN11 levels and the susceptibility of cancer cells to these agents that cause DNA damage (40, 54). Additionally, decitabine can increase SLFN11 expression, rendering cells sensitive to the leading competitive inhibitor, SN-38. TROP2 antibody-drug complexes (ADCs), such as sacituzumab govitecan (SG) antibody-drug conjugate, have shown particularly promising efficacy in triple-negative breast cancer (TNBC). Epigenetic upregulation of both TROP2 and SLFN11 can enhance the therapeutic efficacy of SG (39).

### 3.3 Connection between SLFN12 and cancer

Triple-negative subtype of breast cancer (TNBC) is linked to a poor prognosis and does not have specific targeted treatments available. The Schlafen (SLFN) gene family, especially SLFN12, is essential for regulating the biological processes associated with triple-negative breast cancer (TNBC). Research conducted by AHMED ADHAM RAAFAT ELSAYED et al. (41, 42) have demonstrated that SLFN12 exerts a potentiation effect on the responsiveness of triple-negative breast cancer (TNBC) to DNA-damaging therapies, which is achieved in part through the suppression of CHK1/2 phosphorylation. Enhanced sensitivity has been observed to potentially enhance survival outcomes in Triple Negative Breast Cancer (TNBC) patients characterized by elevated levels of SLFN12 expression, proposing that the expression levels of SLFN12 be considered to serve as a predictive indicator for assessing the efficacy of radiotherapy and chemotherapy in TNBC. In investigating the regulation of SLFN expression in TNBC, Savannah R. Brown and colleagues conducted experiments (55) showing that the administration of IFN-α2 results in the upregulation of SLFN5, SLFN11, SLFN12, a homolog of SLFN12, SLFN13, and SLFN14 in triple-negative breast cancer (TNBC) cell lines, concurrently leading to a decrease in cellular proliferation. The discovery holds promising potential for the advancement of precision treatment strategies in Triple Negative Breast Cancer (TNBC).

SLFN12 is also known to mediate the differentiation of intestinal epithelial cells, as well as prostate and breast cancer cells (23). Jonathan Pacella et al. demonstrated through experiments that SLFN12 improves the prognosis of lung adenocarcinoma, at least in part, by slowing proliferation via the c-Myc pathway (43). The study further suggests that SLFN12 and its downstream effectors may serve as valuable targets for future precision drug design in lung adenocarcinoma treatment.Like other members of the family, it also participated in the HIV intervention process.Schlafen 12 limits HIV-1 latency reversal by imposing a codon-usage-dependent post-transcriptional block in CD4+ T cells (56).

### 3.4 Connection between SLFN13 and cancer

The biochemical cleavage of tRNA and rRNA molecules is a crucial and conserved step in translational control that helps cells overcome various environmental stresses. SLFN13, as a tRNA/rRNA-targeting endoribonuclease, assumes a pivotal function in this process and its knockout significantly reduces cellular resistance to HIV (8).

Based on information from “The Cancer Genome Atlas,” the expression of SLFN13 is reduced in pulmonary squamous cell cancer and rectal cancer, but increased in pancreatic adenocarcinoma and krenal cell cancer (57).

Glioblastoma (GBM) is characterized by its invasive nature and association with dormancy. During the TMZ-promoted dormancy of GBM, several genes are regulated. SLFN13 is involved in TMZ-promoted dormancy and has been shown to be potentially linked to stemness in GBM (44) This suggests that targeting SLFN13 might enhance the antitumor efficacy of TMZ.

### 3.5 Connection between other SLFN family members and cancer

To investigate the expansive function of SLFN in cancer, studies involving mouse models have demonstrated that SLFN2 and SLFN3 regulate tumor development in malignant cutaneous melanoma and renal tumors by modulating cell multiplication (58). Research on SLFN family members, particularly their involvement in various diseases and immune system infiltration, highlights their potential significance in cancer therapy. SLFN3, for instance, is closely linked with the differentiation process of gut epithelial tissues (59). In colon cancer cells that exhibit resistance to FOLFOX and are enriched with cancer stem cells, SLFN3 expression suppresses several malignant characteristics. These include inducing differentiation, reducing the formation of tumor spheres and colonospheres, decreasing pharmacokinetic transporter function, and inhibiting autocrine-stimulated proliferation. Consequently, the expression of SLFN3 may enhance the susceptibility of colon cancer stem cells to chemotherapeutic agents (60). In the study conducted by Sulaiman Sheriff et al., mouse SLFN4 and its human counterpart SLFN12L were identified as markers of a population of cells that travel to the stomach in response to Helicobacter pylori infection and later gain the functionality of myeloid-derived suppressor cells (MDSCs), the gastric metaplasia process commences with a series of intricate biological transformations (23). Further investigations demonstrated that the deletion of SLFN4 in mice, or inhibition of SLFN4 through sildenafil treatment post-H. pylori infection, both significantly reduced H. pylori-induced gastric epithelial metaplasia (61).

## 4 Conclusion

We have identified that Schlafen (SLFN) proteins act as a key player in tumorigenesis, advancement, and treatment. Regarding breast cancer patients with aggressive disease and poor tolerance to chemotherapy, assessing and increasing the expression of SLFN5 may be essential. On one hand, SLFN5 can inhibit epithelial-mesenchymal transition (EMT), slowing the malignant behavior of the disease; on the other, it suppresses tumor cell proliferation. This raises the question of whether modulating SLFN5 expression might offer a viable therapeutic strategy for such patients. Furthermore, the inhibition of SLFN12L has been shown to alleviate Helicobacter pylori (HP)-induced gastric metaplasia, suggesting potential support for clinical treatments where HP eradication is challenging.

Previous studies have demonstrated that the roles of the SLFN family are not universal, and their interaction networks in various diseases are highly complex. Each SLFN protein has distinct mechanisms and functions across different tumors, indicating the need for further exploration of their expression patterns, prognostic significance, and therapeutic potential in specific tumor subtypes. For example, SLFN5 and SLFN12 exhibit positive feedback in breast cancer, while SLFN5, SLFN11, and SLFN13 all play roles in HIV-1 regulation. This raises the intriguing possibility that coordinated regulation of these proteins could provide significant therapeutic benefit, potentially amplifying the impact of interventions targeting disease progression.

The prospect of utilizing SLFN family members as non-invasive preoperative biomarkers for cancer is promising, although substantial experimental evidence is still required. Given the close association between Schlafen proteins and tumor-infiltrating immune cells and immune checkpoints, along with their confirmed role in modulating chemotherapy sensitivity and resistance, assessing their expression and regulatory levels could provide new avenues for combining chemotherapy with immunotherapy. This approach could pave the way for personalized and precision-based treatment strategies. However, whether this advantage can be successfully translated into clinical applications, extending patient survival without introducing long-term adverse effects, requires comprehensive and multi-faceted experimental validation.

In summary, the SLFN family represents a promising group of proteins with significant implications in disease biology, particularly cancer, and warrants further investigation to uncover their full therapeutic potential.
